# Bacterial colonization of the stomach and duodenum in a Swedish population with and without proton pump inhibitor treatment

**DOI:** 10.1002/jgh3.12265

**Published:** 2019-10-01

**Authors:** Jorge Alberto Arroyo Vázquez, Claes Henning, Per‐Ola Park, Maria Bergström

**Affiliations:** ^1^ Department of Surgery South Älvsborg Hospital Borås Sweden; ^2^ University of Gothenburg, Sahlgrenska Academy Gothenburg Sweden; ^3^ Department of Microbiology South Älvsborg Hospital Borås Sweden

**Keywords:** bacteria, gastric bacterial flora, gastroscopy, perforated ulcers, proton pump inhibitor, transgastric interventions, urease

## Abstract

**Background and Aim:**

Microbial contamination of the abdominal cavity is a serious concern during transgastric endoscopic interventions and perforations, particularly in patients who have inhibited gastric acid secretion due to treatment with proton pump inhibitors (PPIs).

The aim of this study was to investigate the gastric and duodenal bacterial flora in patients with and without PPI treatment.

**Methods:**

Patients referred for gastroscopy, without recent antibiotic treatment, were eligible for inclusion. Use of PPIs was recorded. Samples for bacterial culturing were obtained from the antrum of the stomach and from the duodenal bulb through a gastroscope. Positive cultures were examined for bacterial types and subtypes. Biopsies were taken in the antrum for urease test to detect *Helicobacter pylori*.

**Results:**

Bacterial cultures from the stomach were obtained from 103 patients, and duodenal samples were also cultured from 49 of them, for a total of 53 patients with PPI use and 50 patients without. Positive gastric cultures were found in 42 of 53 patients with PPI use and in 13 of 50 without (*P* < 0.0001). Duodenal cultures were positive in 20 of 24 with PPI and 8 of 25 without (*P* < 0.0001). The most commonly identified bacterial species were oral strains of *Streptococcus*, followed by *Neisseria* and *Haemophilus influenzae*. Of 103 patients, 10 had a positive urease test, indicating *H. pylori* infection, 1 with PPI and 9 without.

**Conclusions:**

Bacterial growth in the stomach and duodenum is more common in patients with PPI treatment. The dominating bacterial species found in the stomach and duodenum originates from the oropharynx.

*Clinical trials registry*: Trial registration number 98041 in Researchweb (FoU in Sweden).

## Graphical abstract

Bacterial cultures from the stomach were obtained from 103 patients admitted for routine gastroscopy; 53 patients had ongoing proton pump inhibitor (PPI) treatment. Positive cultures were significantly more common in patients with PPI (*P* < 0.0001). Most common bacterial species were oral strains of *Streptococcus*, followed by *Neisseria* and *Haemophilus influenzae*, resembling oral flora.

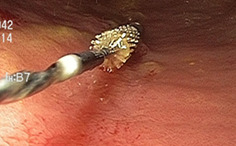

## Introduction

Bacterial contamination of the abdominal cavity may occur during transgastric endoscopic interventions, during gastric surgery, or after perforated peptic ulcers. Human gastric content has been believed to be sterile due to its low pH, suggesting that perforated ulcers resemble clean‐contaminated cases.^1^

Few bacteria can grow at the normal low‐pH conditions of the stomach, apart from *Helicobacter pylori*. A reduction of gastric acid secretion has been shown to induce hypochlorhydria or even achlorhydria, resulting in rising pH, thus increasing the gastric susceptibility for bacterial colonization.^2^, ^3^ A gastric pH above 4 has been shown to allow bacterial colonization of the stomach.^4^

Previous studies of the human gastric bacterial flora have mainly been carried out before the introduction of acid‐decreasing medication, such as histamine H_2_‐receptor antagonists and proton pump inhibitors (PPIs). In a recent pediatric study by Rosen *et al*., a significant difference in gastric bacterial colonization was found between PPI users and nonusers.^5^ There are good reasons to believe that the adult gastric flora of today is influenced by the common use of PPIs. The use of PPIs in Sweden, as measured in daily doses per 1000 inhabitants, shows an 88% increase during the period of 2006–2016 according to The National Board of Health and Welfare in Sweden.^6^

The indications for, and the choice of, prophylactic antibiotics during transgastric endoscopic interventions, gastric surgery, and gastric perforations are subjects of ongoing debate. For better selection of treatment and prophylaxis, further characterization of the gastric flora is needed, taking the modern use of PPIs into account. This would provide a basis for better targeting, lower health‐care costs, and less environmental impact.

The aim of this study was to investigate and characterize the gastric and duodenal bacterial flora in a Swedish population with and without PPI treatment.

## Methods

This study was approved by the Regional Ethics Committee of Västra Götaland, Sweden (Dnr 054‐11) and registered in Researchweb, with trial registration number 98041. Patients referred to the endoscopy unit for gastroscopy were approached and assessed for eligibility. Patients with no PPI intake during the recent 3 months or patients with continuous PPI intake for at least 1 month were included (referred to as “PPI use”). Exclusion criteria were: age < 18 years, antibiotic intake within 3 months prior to gastroscopy, known altered gastric anatomy, on‐demand PPI intake, ongoing immune‐modifying treatment, need for language translation, and nonautonomous patients unable to give consent. Informed consent was obtained by the endoscopist prior to the examination. If a suspected malignancy was found during the procedure, the patient was excluded.

**Figure 1:**
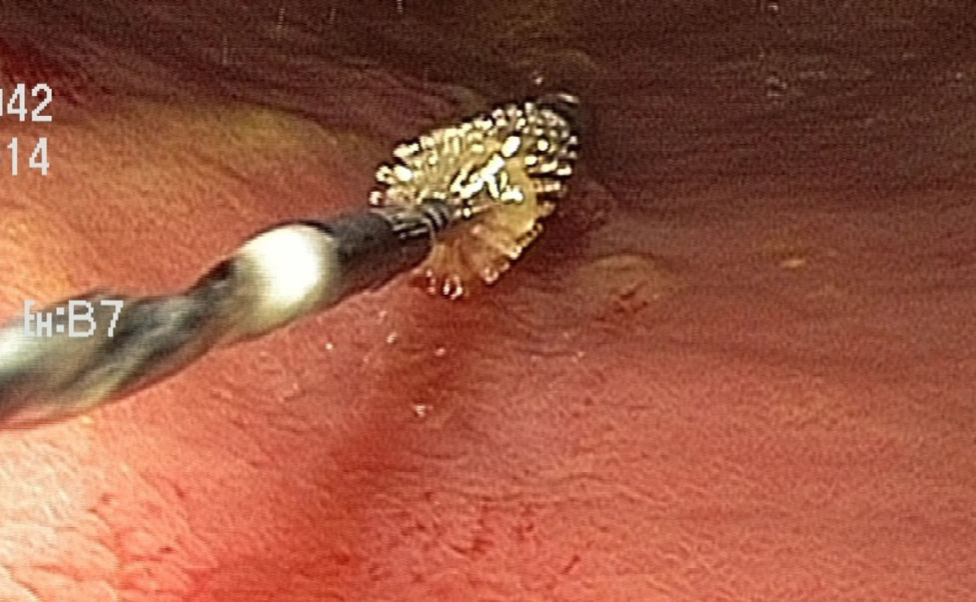
Samples for cultures were obtained using a single‐use cytology brush.

Local preprocedural routines of the endoscopic unit were followed: no intake of solid food for 6 h and no liquids for 6 h prior to the gastroscopy. The examination was performed with the patient in the left lateral decubitus position. Gastroscopy was performed by an endoscopically experienced surgeon. During introduction of the gastroscope into the stomach, no suction was performed. With the gastroscope in the fundic area, the working channel was flushed with 5 mL of saline solution. The scope was then moved to the antrum where a covered cytology brush was used for brushing and sampling from the mucosa. The brush was brought down the working channel inside its plastic cover; was then exposed during sampling; and was retracted inside the cover after sampling and brought up with the brush protected, minimizing contamination. A routine single‐use cytology brush was used (Olympus BC‐17W) (Fig. 1). During the second half of the study (pat no 64‐114), brush samples were also obtained from the duodenal bulb, using a second cytology brush.

After the endoscopic examination and before the gastroscope was retrieved, a biopsy was taken from the gastric antrum using standard biopsy forceps (Olympus EndoJaw FB‐220K) to detect *H. pylori* using a urease test (HelicotecUT Plus, Strong–biotech Corporation).

The brushes used for sampling were cut into tubes with sterile 0.9% saline, were brought to the hospital laboratory for microbiology, and were cultured within 4 h. Culture was performed on GC agar (GC agar‐acumedia with 2% hemoglobin and 1% isovitalex), blood agar (Blood agar base no 2‐Oxoid with 5% horse blood), and anaerobic agar with two 10 μg gentamicin discs (Fastidious anaerobic agar‐acumedia with 4% human blood).

GC agar was incubated in 6% CO_2_, blood agar in normal air, and anaerobic agar in an anaerobic box (N_2_ with 10% H_2_ and 10% CO_2_) at 36°C for 48 h. GC agar and blood agar were inspected daily and anaerobic agar after 2 days. Matrix‐Assisted Laser Desorption/Ionization‐Time Of Flight (MALDI‐TOF) (VITEK‐MS, Biomérieux) was used to identify different bacterial colonies.

The samples collected from the antrum of the stomach were cultured and analyzed for common Gram‐negative bacteria (*Escherichia coli*, *Klebsiella*, *Enterobacter*, Proteus, *Pseudomonas*) and Gram‐positive bacteria (*Staphylococcus*, *Streptococcus*), common bacteria of the oral cavity (alpha‐streptococci, *Neisseria*, *Haemophilus*, pneumococci), and anaerobes (*Bacteriodes*, clostridia). The cut‐off value for the cultures was 50 CFU/mL.

### *Statistics*

Values are given as median and range. Comparisons between groups were performed using nonparametric tests, the Mann–Whitney U test for unrelated data, and the Chi‐square test for nominal data. All statistics were processed using the IBM SPSS 24 statistics software. Differences were considered statistically significant at *P* < 0.05.

## Results

### *Demographics and PPI intake*

**Figure 2:**
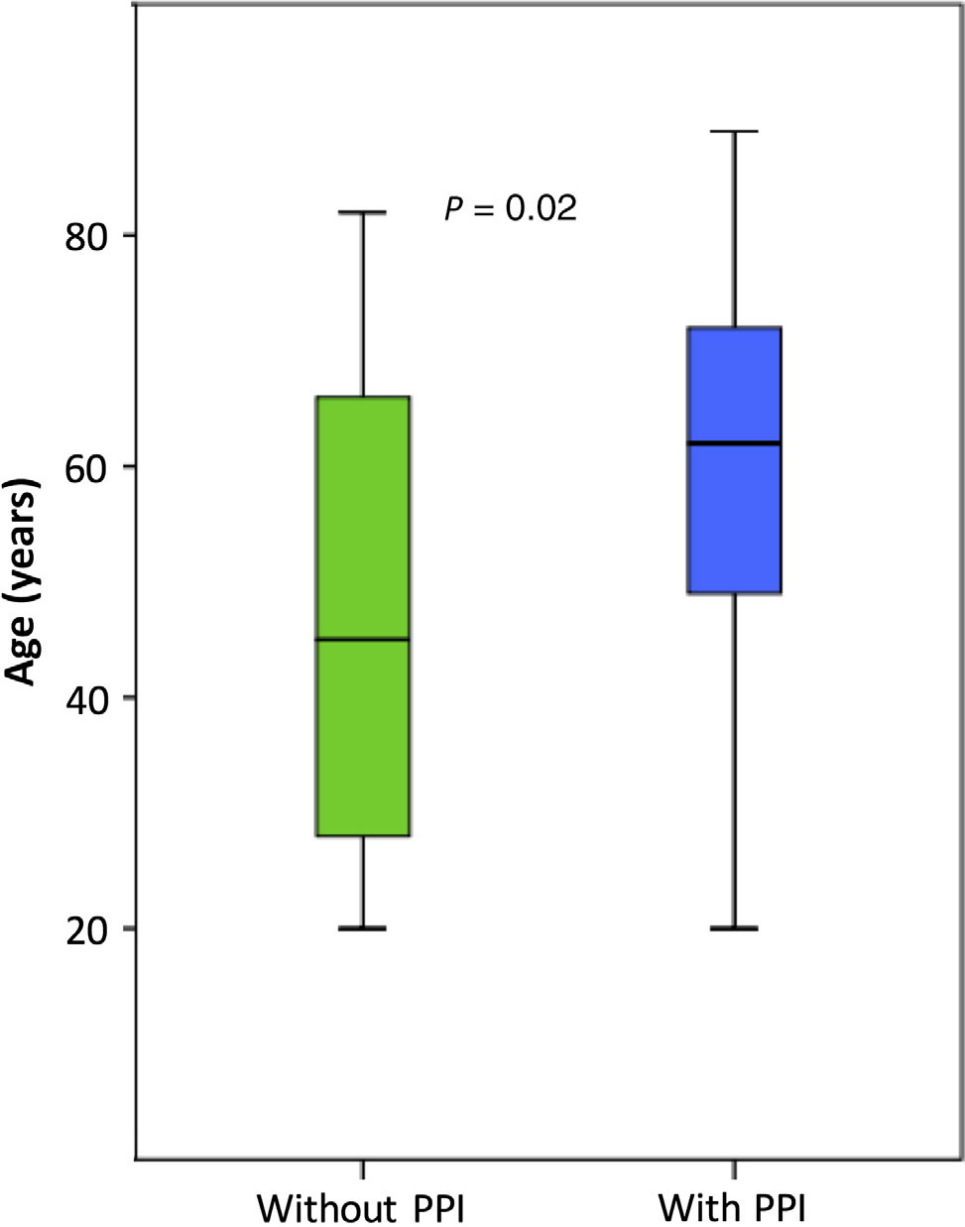
Proton pump inhibitor (PPI) users were significantly older than non‐PPI users, *P* = 0.02.

A total of 114 patients were included in the study. Eleven were excluded before analysis due to missed exclusion criteria (four patients were excluded due to antibiotic intake, five due to on‐demand PPI intake, and two due to previously unknown surgically altered anatomy). After exclusion, 103 patients remained eligible for analysis. The main indications for gastroscopy were reflux symptoms (31 patients) followed by nausea/vomiting (20), abdominal pain (19), anemia/bleeding (15) and 18 miscellaneous indications. Of the 103 patients, 53 had continuous PPI treatment. Median age was 62 years (20–89) in the PPI group and 45 years (20–82) in the non‐PPI group (*P* = 0.02) (Fig. 2). There was no difference in gender distribution between the groups (overall 59% female).

### *Cultures*

**Figure 3:**
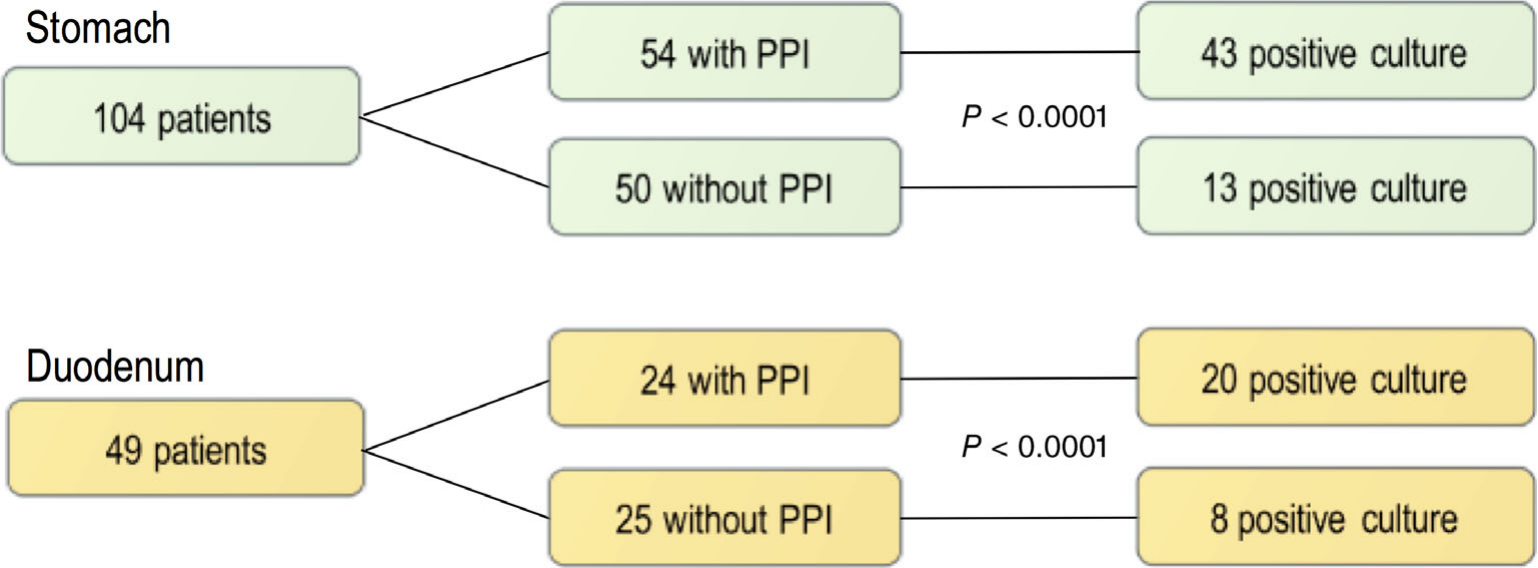
Number of included patients for the two sampling locations, proton pump inhibitor (PPI) usage, and bacterial cultures.

**Figure 4:**
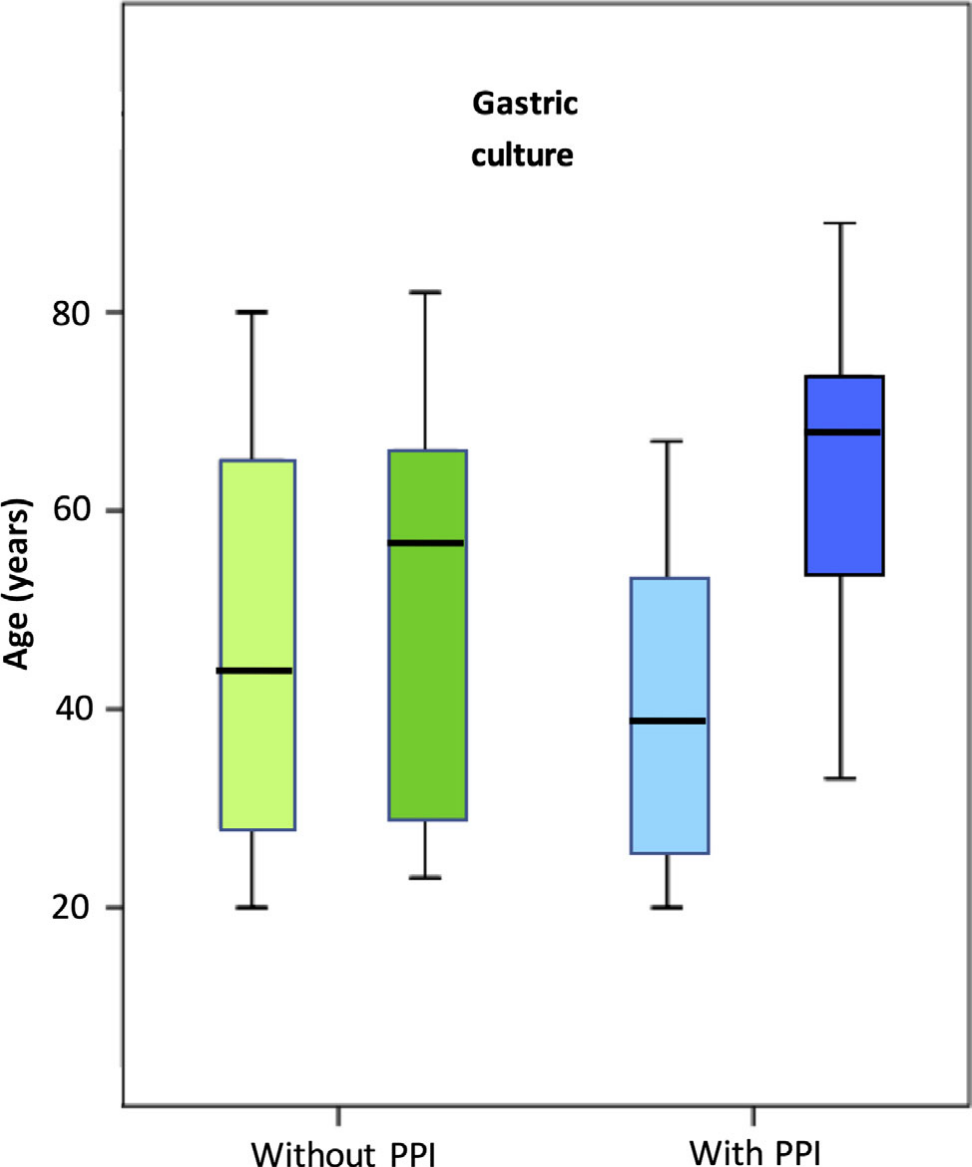
Age and gastric culture results in patients with and without proton pump inhibitor (PPI) usage. (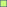), Negative; (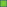), positive; (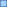), negative; (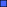), positive.

Of the 103 patients, 55 had a positive gastric culture, and of the 49 patients with parallel duodenal sampling, 28 had a positive culture. A total of 21 patients had positive cultures from both the stomach and duodenum. In the PPI group, 42 (79%) of 53 patients had a positive gastric culture in comparison with 13 of 50 (26%) in the non‐PPI group, showing a significant difference (*P* < 0.0001). Duodenal sampling showed a positive culture in 20 of24 (83%) in the PPI group and in 8 of 25 (32%) in the non‐PPI group, which was also significantly different (*P* < 0.0001) (Fig. 3). There was no difference in age distribution between positive and negative cultures among patients without PPI. However, in the group with PPI treatment, patients with a positive culture were significantly older than those with a negative culture (*P* = 0.001) (Fig. 4).

**Figure 5:**
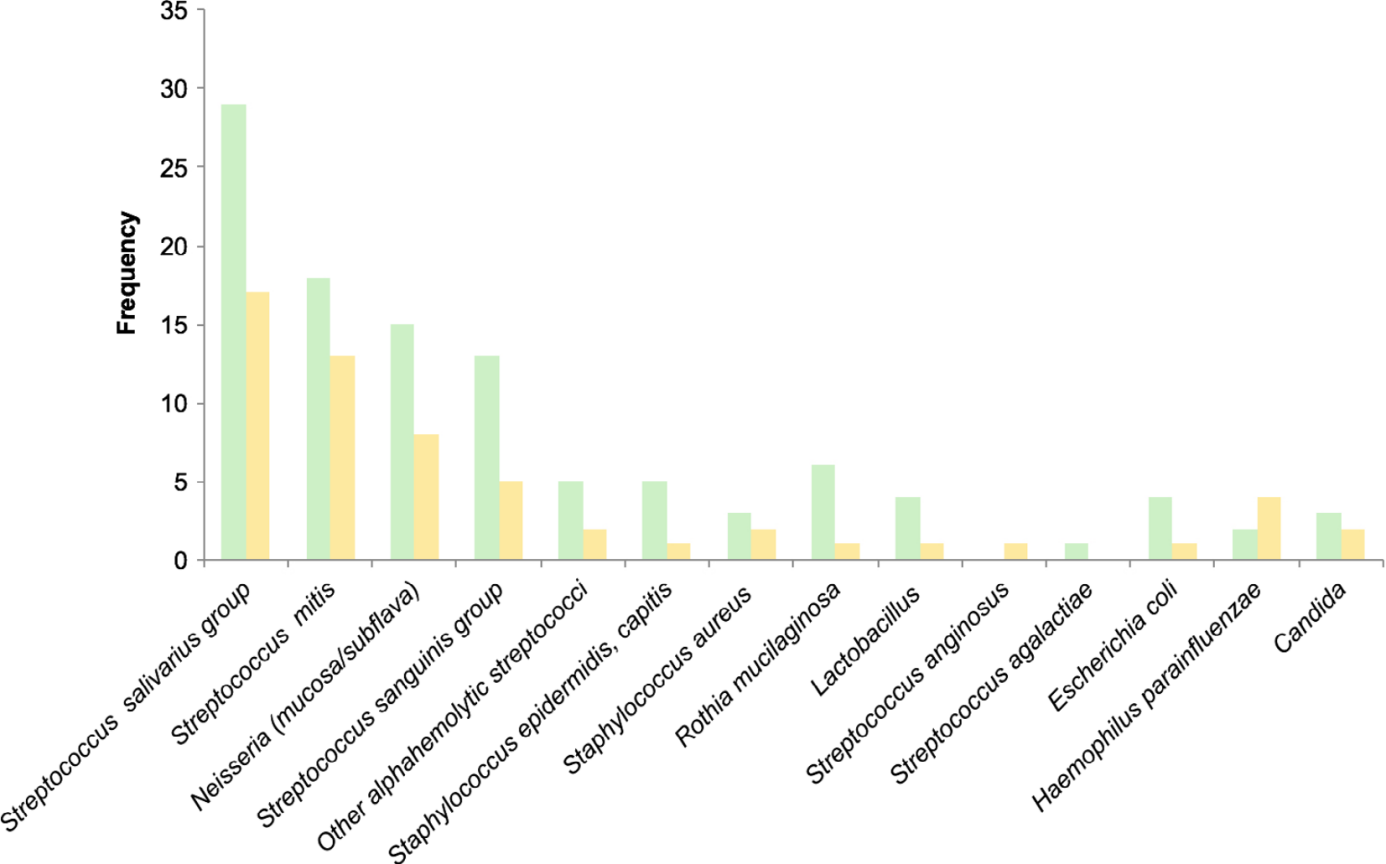
Bacterial species found in the stomach and duodenum. (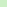), Stomach; (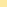), duodenum.

The most commonly identified bacterial species were oral streptococcal strains of several oral subtypes, such as *Streptococcus salivarius* and *Streptococcus mitis*, followed by *Neisseria* species (*mucosa/subflava*) and *Streptococcus parasanguinis* and *Staphylococcus epidermidis*, *Staphylococcus capiti*, *Staphylococcus aureus*, *Rothia mucilaginosa*, *Lactobacillus* (*catharralis*, *ghassesi*), alpha‐ streptococci, *Streptococcus vestibularis*, and *E. coli* (Fig. 5).

**Figure 6:**
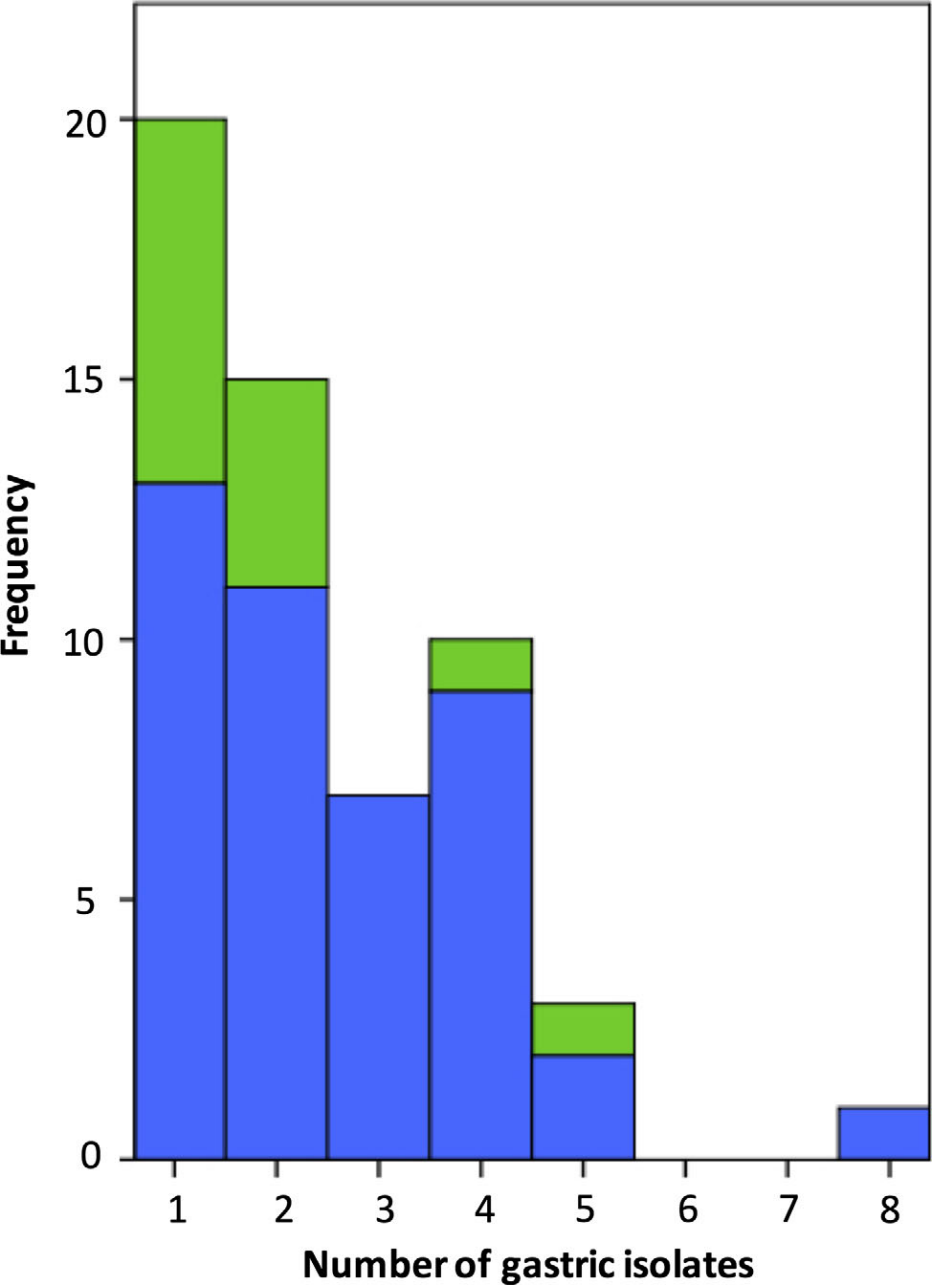
Number of gastric isolates in patients with positive culture. (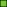), Without proton pump inhibitor (PPI); (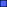), with PPI.

Among all 55 patients with a positive culture from either the stomach, the duodenum, or both, 36 (65%) showed colonization with bacteria of more than one strain^2^, ^3^, ^4^, ^5^, ^6^, ^7^, ^8^ (Fig. 6).

A positive urease test for *H. pylori* was found in 10 of the 103 patients (10%), three women and seven men (ns). Of these 10 patients, 9 were not on PPI treatment.

## Discussion

The current study shows that bacteria are significantly more present in the stomach and the duodenal bulb in patients on continuous PPI treatment than in those without PPIs, indicating that reduced secretion of gastric acid changes the conditions for bacterial survival or colonization. Current results also indicate that most of the bacteria found in the stomach resemble those commonly detected in the oropharyngeal cavity.

PPI medication increases the gastric pH by inhibiting acid secretion. A pH > 4 has been shown to allow bacterial colonization of the stomach, either by allowing bacterial colonization and growth or by a decreased antibacterial effect.^3^, ^4^ In a previous study where achlorhydria was induced, even in a short duration, overgrowth of Gram‐positive organisms resembling the oropharyngeal flora was shown.^7^ Gastric bacterial colonization with oropharyngeal flora, as shown in the current study, might be explained by the lack of destruction of bacteria swallowed from the oropharynx rather than bacterial replication in the stomach.^2^

The *oropharyngeal bacteria* are not locally pathogenic in the stomach or duodenum. Bacterial contamination of the abdominal cavity may occur during both elective transgastric endoscopic interventions and gastric surgery and after acute gastrointestinal perforations. Choosing the right initial empiric antibiotic for patients in need of emergency surgery, including perforated peptic ulcers, improves their outcome.^8^

For acute stomach and duodenal perforations, current recommendations for prophylactic antibiotics formulated by the Infectious Diseases Society of America (IDSA) advise the following: in the absence of acid‐reducing therapy or malignancy and when source control is achieved within 24 h, prophylactic anti‐infective therapy directed at aerobic Gram‐positive cocci is recommended. However, if surgery is delayed or the presence of therapy‐reducing gastric acidity is detected, antimicrobial therapy to cover mixed flora, such as meropenem, piperacillin/tazobactam, or ciprofloxacin, is needed.^9^ The current study indicates that an early abdominal bacterial contamination mainly consists of orally derived bacteria, mostly streptococci, which is why antibiotics with a narrow spectrum might be sufficient.

Studies from the 1990s and earlier have shown that gastric acidity decreases with age.^10^ One reason was the higher prevalence of atrophic gastritis in the elderly, leading to decreased acid production. Atrophic gastritis is related to *H. pylori* infections,^11^ a condition in decline in Scandinavia during recent decades.^12^ Consequently, the prevalence of atrophic gastritis is decreasing in the elderly.^13^ This explains that recent studies of the aging population show preserved gastric acid production in *H. pylori*‐negative subjects.^11^, ^14^ In the current study, age did not correlate to bacterial colonization among patients without PPI. However, among patients with PPI, increasing age correlated to positive cultures, a finding we cannot explain.

In our study, 10% of the subjects showed a positive urease test, indicating the presence of *H. pylori*. This proportion is somewhat lower than the current Swedish prevalence, proposed to be 15–20% and increasing with age.^12^ One factor influencing the low rate of urease positivity in our study population might be that 50% were continuous PPI users. Another factor is that sampling was only performed in the antrum and not in both the antrum and the corpus of the stomach, as proposed by several authors.^15^ The presence of *H. pylori* in the stomach is not in itself relevant to the aims of the present study as these bacteria do not contribute to surgical infectious complications. However, as the presence of *H. pylori* is known to increase stomach pH, allowing other bacteria to colonize and grow,^16^ their presence might still be of relevance regarding surgical infections.

A limitation of this study is that no bacterial quantification was carried out, making it difficult to evaluate the clinical significance of the bacterial findings.

In conclusion, this study indicates that gastric and duodenal bacterial colonization is more common among patients with PPI medication and in this group increasing with age. Given the species that were identified and considering the increasing frequency of antibiotics resistance,^17^ prophylaxis with antibiotics that cover the oropharyngeal flora should be sufficient for patients undergoing transgastric endoscopic interventions or gastrointestinal perforations.
